# State of art fusion-finder algorithms are suitable to detect transcription-induced chimeras in normal tissues?

**DOI:** 10.1186/1471-2105-14-S7-S2

**Published:** 2013-04-22

**Authors:** Matteo Carrara, Marco Beccuti, Federica Cavallo, Susanna Donatelli, Fulvio Lazzarato, Francesca Cordero, Raffaele A Calogero

**Affiliations:** 1 University of Torino, Bioinformatics & Genomics unit, Molecular Biotechnology Center, Via Nizza 52, 10126 Torino, Italy; 2 University of Torino, Department of Computer Science, Corso Svizzera 185, 10149 Torino, Italy; 3 University of Torino, Unit of Cancer Epidemiology, Department of Biomedical Sciences and Human Oncology, Via Santena 7, 10126 Torino, Italy

## Abstract

**Background:**

RNA-seq has the potential to discover genes created by chromosomal rearrangements. Fusion genes, also known as "chimeras", are formed by the breakage and re-joining of two different chromosomes. It is known that chimeras have been implicated in the development of cancer. Few publications in the past showed the presence of fusion events also in normal tissue, but with very limited overlaps between their results. More recently, two fusion genes in normal tissues were detected using both RNA-seq and protein data.

Due to heterogeneous results in identifying chimeras in normal tissue, we decided to evaluate the efficacy of state of the art fusion finders in detecting chimeras in RNA-seq data from normal tissues.

**Results:**

We compared the performance of six fusion-finder tools: FusionHunter, FusionMap, FusionFinder, MapSplice, deFuse and TopHat-fusion. To evaluate the sensitivity we used a synthetic dataset of fusion-products, called positive dataset; in these experiments FusionMap, FusionFinder, MapSplice, and TopHat-fusion are able to detect more than 78% of fusion genes. All tools were error prone with high variability among the tools, identifying some fusion genes not present in the synthetic dataset. To better investigate the false discovery chimera detection rate, synthetic datasets free of fusion-products, called negative datasets, were used. The negative datasets have different read lengths and quality scores, which allow detecting dependency of the tools on both these features. FusionMap, FusionFinder, mapSplice, deFuse and TopHat-fusion were error-prone. Only FusionHunter results were free of false positive. FusionMap gave the best compromise in terms of specificity in the negative dataset and of sensitivity in the positive dataset.

**Conclusions:**

We have observed a dependency of the tools on read length, quality score and on the number of reads supporting each chimera. Thus, it is important to carefully select the software on the basis of the structure of the RNA-seq data under analysis. Furthermore, the sensitivity of chimera detection tools does not seem to be sufficient to provide results consistent with those obtained in normal tissues on the basis of fusion events extracted from published data.

## Background

**Figure 1:**
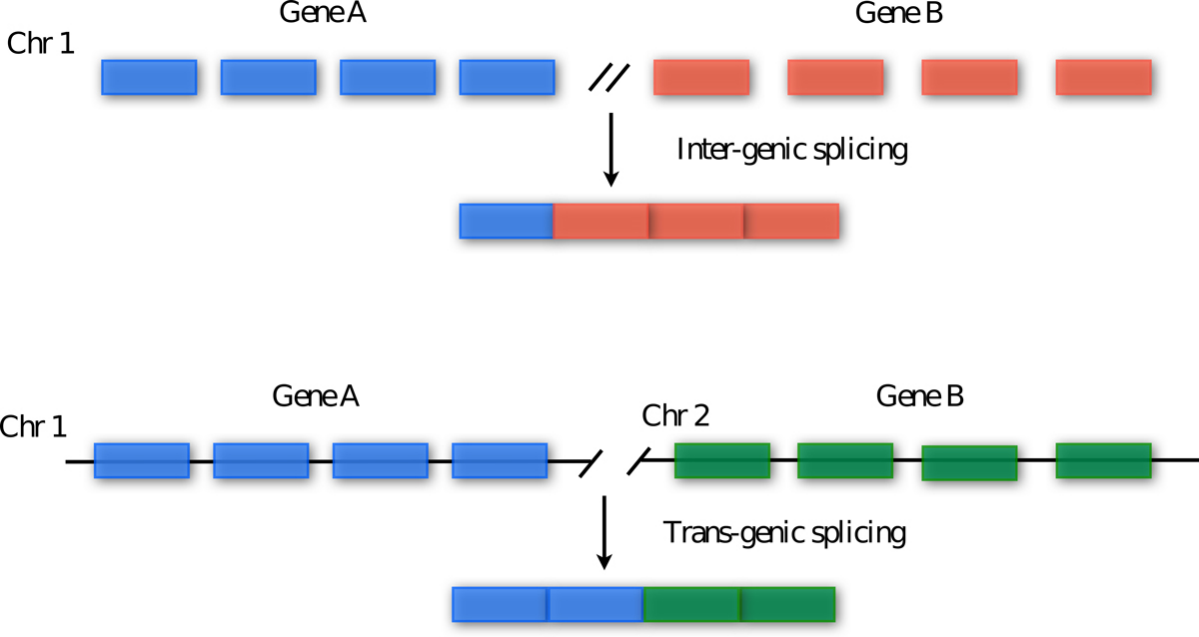
**Events involved in chimeras formation**. Chimeras, not due to a genomic pathological-associated rearrangement, may originate from two separate events: *intergenic splicing *and *transgenic splicing*. An *intergenic splicing *event combines exons from two adjacent genes of the same chromosome, while a *transgenic splicing *event combines exons from two gene locate on different chromosomes.

Sequencing of mRNA transcripts using RNA-seq protocol [1] is becoming the reference method for detecting and quantifying genes expressed in a cell. Although RNA-seq technology is still in the early phase and it has not disclosed completely its potential, http://encodeproject.org/ENCODE/protocols/dataStandards/ENCODE_RNAseq_Standards_V1.0.pdf, it can be used to discover genes created by chromosomal rearrangements. Thus, this technology represents an ideal tool for the discovery of fusion genes, formed by breakage and re-joining of two different chromosomes, which are implicated in the development of cancer [2]. However, normal cells seem to be also characterized by *intergenic splicing *and *transgenic splicing*, namely chimera [3]. As shown in Figure 1, *intergenic splicing *refers to a splicing event between two adjacent genes in the genome, while *transgenic splicing *is an event that produces a chimera comprising exons of two genes located on different chromosomes. Chimeras on the basis of EST estimations [4,5] and more recently by RNA-seq [6] were observed in normal tissues. We refer to these approaches as *ab-initio *since the authors rely on genomic data, without additional biological support, to detect fusions. The experiments reported in [6] indicate that at least 4-6% of genes in the genome may be involved in chimera formation, although their prevalence was found to be generally low. Moreover, targeted alignment against artificial exon-exon junctions [6] of single-end reads RNA-seq data, allowed the detection of a significant amount of chimeras in normal colon and brain tissues as well as in primary colon tumors. No overlap could be observed between the results obtained with EST and RNA-seq based approaches [6].

Recently, Frenkel-Morgenstern et al. [7] described a new approach to assess chimeras. We term this procedure as the *knowledge-based approach *since it is based on fusion events extracted from published data. The authors studied 7,424 putative human chimeric RNAs [8] and detected the expression of 172 chimeric RNAs in 16 human tissues (Illumina Body Map 2.0, GSE30611) using high throughput RNA sequencing, mass spectrometry experimental data, and functional annotations.

### Fusion finder algorithms

In the last two years many chimera-detection tools have been developed and published. To the best of our knowledge, ChimeraScan [9], deFuse [10], FusionFinder [11], FusionHunter [12], FusionMap [13], MapSplice [14], ShortFuse [15], TopHat-Fusion [16] are the most commonly used tools for chimera detection. ChimeraScan and ShortFuse were not considered here since their run did not terminate properly during the preliminary testing phase. Before describing fusion finder algorithms, we introduce the terms used in the rest of the paper.

RNA-seq experiments provide a set of short reads that can be in two forms: single-end or paired-end. In the latter case both the forward and reverse template strands of DNA fragment are sequenced. According to the identification of fusion boundary (the nucleotide coordinates defining the breakpoint of both genes involved in the fusion) it is possible to observe two contexts: read *spanning *or read *encompassing*. Encompassing reads harbor a fusion boundary and each read maps on a different gene of the fused gene couple, while in spanning reads one mate overlaps with a fusion event, while the corresponding paired-end mate matches with one of the two genes involved in the chimera.

We have categorized the fusion detection algorithms into two classes: the *fragment-based approach *and the *pseudo-reference based approach*.

In the *fragment based approach *input reads are split into fragments, which are aligned with respect to reference (whole genome or transcriptome). The mapped fragments are then used to build a list of putative chimeras that undergo through a further selection by means of various types of filters. This category includes the following tools: FusionFinder, FusionMap, MapSlice, deFuse. *Pseudo-reference based approaches *use candidate chimeras, obtained from the previous mapping phase, to generate a new pseudo reference for chimeras detection. The fusion events resulting from the latter step are further filtered to reduce false positive. TopHat-Fusion and FusionHunter are the tools included in this category.

In this paper, we focus on fusion finder algorithms for *ab-initio *processes. Between those algorithms, FusionMap has shown the best compromise between sensitivity and sensibility. Its results have been also compared with results obtained by the *knowledge-based approach *presented in Frenkel-Morgenstern's paper.

## Results

### Evaluating the sensitivity of fusion-finder algorithms

To compare the sensitivity of fusion-finder algorithms we used a synthetic dataset provided as part of the release of the FusionMap software, and we used it as positive dataset.

This dataset encompasses a total of 50 chimeras, supported by a different coverage. In particular, the chimeras are characterized by a number of supporting paired-end reads ranging from 9 to 8852. The analysis of the positive dataset revealed that FusionFinder is the most sensitive tools. Based on the sensitivity, the tools can be ordered as FusionFinder > TopHat-Fusion = FusionMap > MapSplice > deFuse > FusionHunter as reported in Table 1. The table also reports the number of false chimeras detected by each tool, i.e. identification of fusion genes not present in the positive synthetic set. When ranked by the false discovery rate the order changes as follows: deFuse = FusionHunter < FusionMap < FusionFinder < MapSplice < < TopHat-Fusion. FusionMap thus appears to provide the best compromise between sensitivity and false discovery rate.

**Table 1:** Chimera detection performances on positive dataset encompassing 50 synthetic fusion events

Tool	Sensitivity (%)	False discovery rate
FusionFinder	82 (41/50)	10
FusionMap	80 (40/50)	6
TopHat-fusion	80 (40/50)	73
MapSplice	78 (39/50)	23
deFuse	64 (32/50)	4
FusionHunter	40 (20/50)	4

In parenthesis are given the number of fusions

The sensitivity of each tool is given by the number of chimeras detected by each tool divided for the total number of chimeras in the positive dataset. False discovery rate is given as the total number of chimeras detected that do not match any of the positive 50 chimeras.

**Figure 2:**
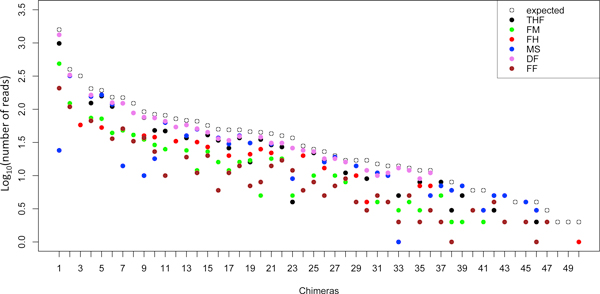
**Chimeras detection in the positive dataset**. The expected number of reads (open circle) associated to each chimera in the positive dataset is shown together with the reads detected by the six different fusion finders. THF: TopHat-fusion, FM: FusionMap, FH: FusionHunter, MS: MapSplice, DF: deFuse, FF: FusionFinder.

We have also evaluated the number of supporting reads detected by the six fusion finders on the positive dataset (Figure 2). All six tools detect a number of reads that are lower than the number present in the dataset (expected reads). It is notable that deFuse detects a number of reads near to expectation for fusions supported by more than 18 reads. Also the other tools lose sensitivity in case of a low number of supporting reads, but they are also characterized by a lack of detection for fusion events supported by a high number of reads.

### Evaluating the false discovery rate of fusion finder tools

**Figure 3:**
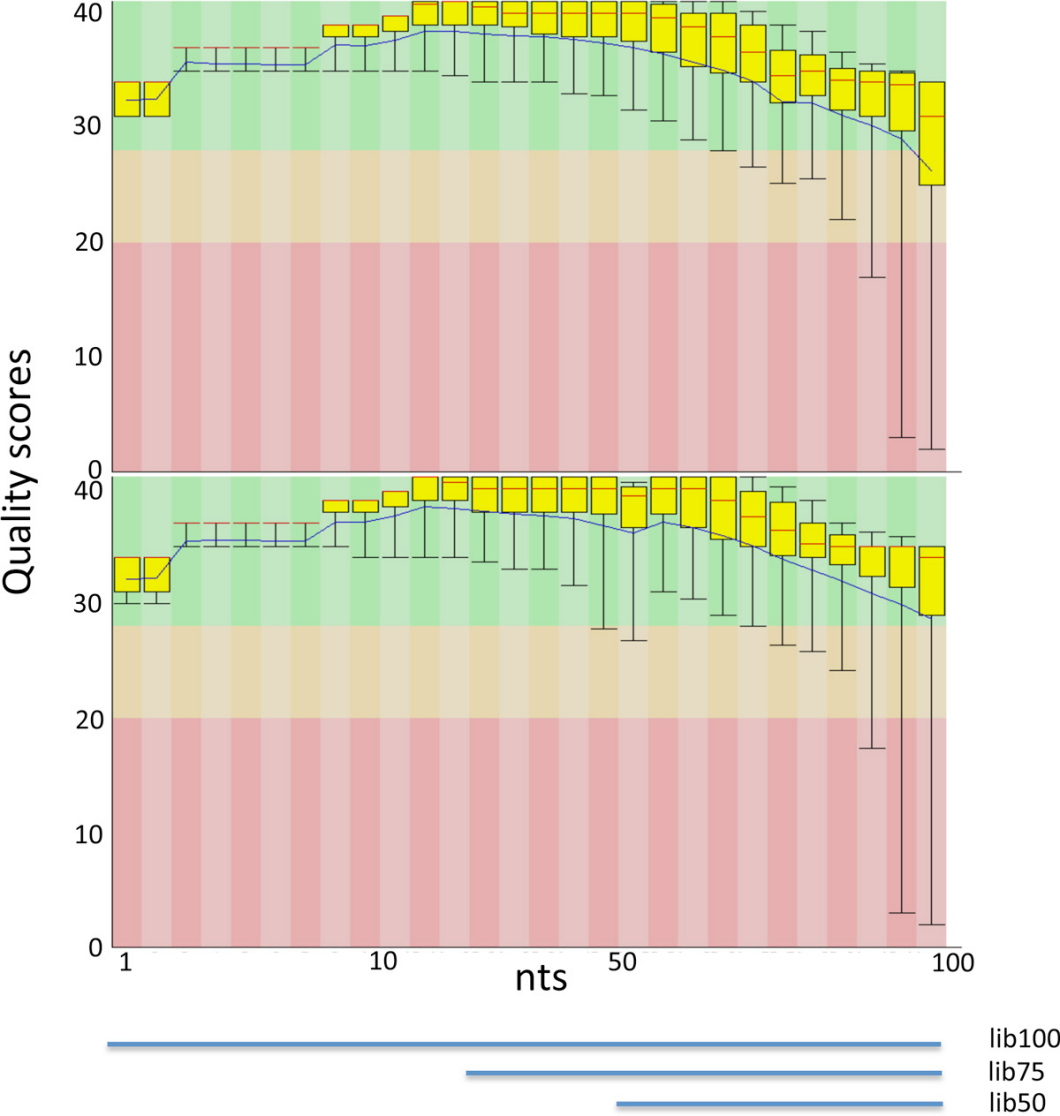
**Distribution of the quality scores associated with lib100_1 and lib100_2**. The same reads generated with BEERS software were associated with two different sets of quality scores. Upper panel: quality scores associated with lib100_1. Lower panel: quality scores associated with lib100_2. The lines in the bottom of the figure indicate the subset of quality scores used for generating the 2 × 50 and 2 × 75 nts fastq files.

**Figure 4:**
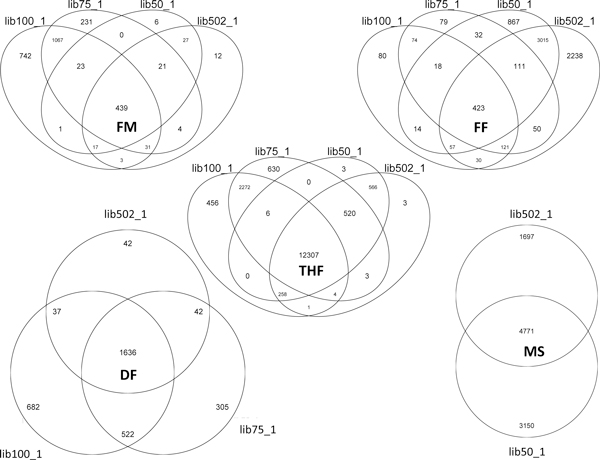
**Venn diagrams of genes detected as part of false chimera in negative datasets**. FM) FusionMap shows a direct dependency of false chimeras with respect to the read length and a limited dependency of false chimera detection on the basis of the quality scores associated with the reads. FF) FusionFinder shows an inverse dependency of false chimeras on the basis of the read length and a strong dependency of false chimera detection on the basis of the quality scores associated with the reads. THF) TopHat-Fusion detects the highest number of false chimeras. Its dependency with respect to read length is quite limited. DF) deFuse shows a direct dependency of false chimeras on the basis of the read length. MS) MapSplice shows a significant dependency of false chimera detection on the basis of the quality scores associated with the reads. FusionHunter is not shown, since it is the only tool that does not detect false chimeras in the negative datasets.

To better understand the detection of false fusion events we constructed a semi-synthetic paired-end dataset composed by 70 million 100 bps reads. The dataset was built using BEERS [17]. BEERS does not simulate quality scores, required by many fusion finder tools, thus we added scores obtained by experiments conducted in our laboratory, giving rise to two paired-end fastq datasets: lib100_1, and lib100_2, associated with two similar sets of quality scores (Figure 3). Different quality score sets led to the evaluation of the effect of quality score on chimera detection. Furthermore, four other datasets, two of 75 bp reads (lib75_1, lib75_2) and two of 50 bp reads (lib50_1, lib50_2), were generated from lib100_1, lib100_2 (Figure 3), to evaluate the effect of read size on the detection of chimera false discovery. FusionFinder, FusionHunter, FusionMap, MapSplice, deFuse, TopHat-Fusion were used to analyze the negative datasets. Table 2 lists the number of false chimeras detected, while Figure 4 shows read length and quality score dependency for genes involved in false fusions. FusionHunter was the only tool that did not detect false chimeras in any of the negative datasets (Table 2). FusionMap and deFuse showed a direct dependency of the number of false chimeras from the read length (Table 2). FusionMap also showed a limited dependency of false chimera detection on the basis of quality scores associated with the reads (Figure 4-FM). In comparison, FusionFinder showed an inverse dependency of false chimera detection from the read length (Table 2) and a strong dependency of false chimera detection on the basis of the read quality scores (Figure 4-FF). TopHat-Fusion detected the highest number of false chimeras, although its dependency with respect to read length and quality score was limited (Figure 2-THF). The results of MapSplice appear to be correlated to the quality scores (Figure 2-MS). According to the false discovery rate, tools can be ranked as: FusionHunter < < FusionMap < FusionFinder < deFuse < < MapSplice < TopHat-Fusion. We also counted the number of reads associated to the false chimeras detected by only five out of six tools, since FusionHunter did not detect any false positive chimera. In the case of TopHat-fusion and MapSplice the median of the supporting reads for false positive was one read for all negative datasets (Additional file 1, THF2 and MS2), but some false fusions were supported by a dozen to hundreds of reads (Additional file 1, THF1 and MS1). A Similar scenario was found for deFuse, with a median of the supporting reads for false positive in the order of 10 reads for all negative datasets analyzed (Additional file 1, DF2). FusionMap and FusionFinder were also characterized by a median of 1 for false positive supporting reads (Additional file 1, FM2, FF2), but in the worst situation false fusions were supported by less than 20 reads for FusionMap, in the lib50 negative dataset (Additional file 1, FM2), and by less than 100 reads for FusionFinder (Additional file 1, FF2).

**Table 2:** False chimera detection

Tool	Lib50_1	lib50_2	Lib75_1	Lib100_1
FusionHunter	0	0	0	0
FusionMap	342	359	1521	2225
FusionFinder	3517	5417	750	666
deFuse	-*	1532	2380	2976
MapSplice	30022	18540	-*	-
TopHat-fusion	60839	60854	122885	112779

*The analysis did not produce the results due to a software error occurring in the handling of an intermediate file.

Number of chimeras detected in datasets free of fusion events (negative datasets). Analysis is performed using different read lengths for the same negative dataset (lib100_1, lib75_1, lib50_1). In case of the 50 nts paired-end reads negative dataset reads were also analyzed considering two different sets of experimental quality scores (lib50_1, lib50_2).

### Searching for chimeras on real dataset with FusionMap

Since FusionMap provided the best compromise between false and true fusions detection, we checked its performance on a real dataset: the Body Map 2.0. We used the 50 bp paired-end dataset and we checked FusionMap results against those presented by Frenkel-Morgenstern [7] on the Body Map 2.0 75 bp single-end dataset. As positive controls we used a subset of the 172 fusion events reported by the authors. We checked these 172 fusion by blasting them with respect to the genome and we ensured that each chimera encompasses genomic regions with the following characteristics: i) genomic regions should not belong to the same gene, ii) each genomic region should not match on multiple chromosomes, iii) each region involved in the fusion should not match on more than two different chromosomal loci. Unexpectedly, only 22 fusion genes, reported in Table 3, exhibit all three characteristics; these events represent the minimal set of positive chimeras, which are expected to be detected in real dataset obtained from normal tissues.

**Table 3:** Genomic locations of genes involved in chimeras detected in Body Map 2.0 in [7]

Fusion EST	EST source	geneA	chrA	startA	endA	geneB	chrB	startB	endB
BE835085	Li paper [21]	MPHOSPH10	chr2	71,357,444	71,377,232	AES	ch19	3,052,908	3,062,964
AF103493	Li paper [21]	IGKJ1	chr2	89,161,398	89,161,435	IGKV1OR22-1	chr22	17,413,617	17,415,543
ENA|AI400677|AI400677.1	chimerDB_ESTs	ZMYM6NB	chr1	35,447,127	35,450,948	ALB	chr4	74,269,972	74,287,129
ENA|AI805048|AI805048.1	chimerDB_ESTs	FXYD3	chr19	35,606,732	35,615,228	ZFYVE19	chr15	41,099,274	41,106,767
ENA|AV722190|AV722190.1	chimerDB_ESTs	PICALM	chr11	85,668,214	85,780,923	SPP1	chr4	88,896,802	88,904,563
ENA|AW206715|AW206715.1	chimerDB_ESTs	RAMP2	chr17	40,913,212	40,915,059	ZNF3	chr7	99,661,653	99,679,371
ENA|AW316925|AW316925.1	chimerDB_ESTs	GNB2	chr7	100,271,363	100,276,792	QSOX1	chr1	180,123,968	180,167,169
ENA|AW627635|AW627635.1	chimerDB_ESTs	LOC100294406	chr2	89,148,206	89,231,927	RBM10	chrX	47,004,617	47,046,214
ENA|BE903629|BE903629.1	chimerDB_ESTs	CSNK2B	chr6	31,633,657	31,637,843	RPL8	chr8	146,015,154	146,017,805
ENA|BG564612|BG564612.1	chimerDB_ESTs	GSTK1	chr7	142,960,522	142,966,222	HP	chr16	72,088,508	72,094,955
ENA|BG978110|BG978110.1	chimerDB_ESTs	PSMB1	chr6	170,844,204	170,862,417	GSTP1	chr11	67,351,066	67,354,124
ENA|BM559993|BM559993.1	chimerDB_ESTs	HLA-E	chr6	30457183	30,461,982	PPFIBP1	chr12	27,677,045	27,848,497
ENA|BM827569|BM827569.1	chimerDB_ESTs	ELOVL5	chr6	53,132,196	53,213,977	CYBA	chr16	88,709,697	88,717,457
ENA|BP419192|BP419192.1	chimerDB_ESTs	FBLIM1	chr1	16,085,255	16,113,084	AKIP1	chr11	8,932,701	8,941,626
ENA|BQ004985|BQ004985.1	chimerDB_ESTs	F2RL1	chr5	76,114,833	76,131,140	COL1A2	chr7	94,023,873	94,060,544
ENA|BQ010435|BQ010435.1	chimerDB_ESTs	CLSTN1	chr1	9,789,079	9,884,550	LAPTM4A	chr2	20,232,411	20,251,789
ENA|BU684515|BU684515.1	chimerDB_ESTs	NDUFA13	chr19	19,627,019	19,639,013	FLNA	chrX	153,576,900	153,603,006
ENA|CD742870|CD742870.1	chimerDB_ESTs	HLA-G	chr6	29,794,756	29,798,899	PPP1R15A	chr19	49,375,649	49,379,319
ENA|CF125182|CF125182.1	chimerDB_ESTs	PICALM	chr11	85,668,214	85,780,923	CPQ	chr8	97,657,499	98,155,722
ENA|DA932721|DA932721.1	chimerDB_ESTs	CD74	chr5	149,781,200	149,792,332	SCARF1	chr17	1,537,152	1,549,041
ENA|T05374|T05374.1	chimerDB_ESTs	SRPRB	chr3	133,502,877	133,540,336	SLC22A23	chr6	3,269,207	3,456,793
EF051633	chimerDB_ESTmRNAs	PICALM	chr11	85,668,214	85,780,923	MLLT10	chr10	21,823,101	22,032,559

The subset of 22 chimeras encompassing only two genes on different chromosomes, extracted from the 172 events validated by Frenkel-Morgenstern, using the 75 nts single-end reads RNA-seq Body Map 2.0 dataset, was used as positive control of the ability of FusionMap to detect chimers in normal tissues RNA-seq data.

The analysis performed with FusionMap detected HLA-E (liver tissue) and SSP1 (ovary tissue) as genes involved in fusions, also identified by Frenkel-Morgenstern [7]. However, the authors detected HLA-E:GSTP1 and RAMP2:SPP1 fusions, whereas in our analysis we detected HLA-E:BCKDHB and SPP1:ABCA10 fusions. We also found other fusions (Table 4), that are not part of the Frenkel-Morgenstern dataset.

**Table 4:** Chimeras detection in Body map 2.0 by FusionMap

Tissue	# of genes involved in chimeras in Body Map 2.0	# of genes also detected in the negative dataset	**# of genes also detected as chimeras in **[7]	**Genes in chimeras **[7]	Chimeras detected by FusionMap	**Chimeras in **[7]
Adipose	74	7	0	-		
Adrenal	60	6	0	-		
Brain	56	10	0	-		
Breast	32	2	0	-		
Colon	15	3	0	-		
Kidney	37	4	0	-		
Heart	18	0	0	-		
Liver	31	2	1	HLA-E	HLA-E:BCKDHB	HLA-E:GSTP1
Lung	46	5	0	-		
Lymph node	37	1	0	-		
Prostate	68	12	0	-		
Skeletal muscle	34	3	0	-		
White blood cells	29	4	0	-		
Ovary	30	3	1	SPP1	SPP1:ABCA10	RAMP2:SPP1

Number of genes detected as part of chimera in Body Map 2.0 50 nts paired-end dataset. Body Map 2.0 50 nts paired-end was generated from the same donors used for the 75 nts single-end dataset used in Frenkel-Morgenstern's paper to validate putative fusion by means of a *knowledge-based approach*. Thus, the two datasets are technical replication of the same mRNA universe.

Table 4 also reports, for each gene involved in the detected chimeras of Body Map, the number of genes that have been falsely detected by FusionMap in the experiment of the negative datasets.

## Discussion

The main goal of this paper was to understand if the main fusion detection software tools, available in the literature, are able to detect chimeras in normal tissue RNA-seq data. To reach our aim, it was essential to understand the behavior of fusion detection software tools. Thus, we evaluated the sensitivity and false discovery rate for six state-of-the-art fusion-finders: FusionHunter, FusionMap, FusionFinder, MapSplice, deFuse and TopHat-fusion.

In our experiments, FusionHunter performed better than all the other tools on the basis of false discovery rate, but had the lowest sensitivity with respect to the others. The behavior of FusionHunter is consistent with two other observations: i) FusionHunter looses all the fusions, in the positive dataset, supported by less than 18 reads, and ii) the median value for false positive chimeras for all tools, excluded FusionHunter, is between 1 to 10 reads. Thus, to reduce the risk of false positive detection, weighting negatively fusions supported by a low number of reads, FusionHunter clearly suffers of a reduced sensitivity. At the same time FusionHunter implements some specific features that make it less sensitive to the discovery of false fusions supported by a high number of reads that are frequently observable in the other fusion detection tools.

Quality scores associated with the datasets affected MapSplice and FusionFinder results. On the other hand, FusionFinder was more sensitive to read length, with a reduction in the false fusion detection rate dependent on a corresponding increase in the read length. Conversely, FusionMap and deFuse performed much better with short reads: the larger the read the higher the number of false positive fusion genes. TopHat-fusion was insensitive to quality score, but it showed the highest false positive discovery rate of the tools tested. With respect to sensitivity, deFuse and FusionHunter, were found to be the least sensitive. The best compromise between sensitivity and specificity was given by FusionMap, which seemed particularly suitable for the analysis of the Illumina normal tissue Body Map 2.0 RNA-seq dataset, since its false fusion detection rate was particularly low in the analysis of negative datasets. Despite the good sensitivity of FusionMap in the test dataset, the analysis of the Body Map 2.0 paired-end reads revealed a low correlation between FusionMap fusions detected in this dataset and fusions detected in the single-end dataset by Frenkel-Morgenstern. An important point to be considered, when comparing the results obtained with the 75 bp reads single-end and the 50 bp reads paired-end Body Map 2.0 datasets, is tissue source origin. The two datasets are generated starting, for each tissue, from the same donor, therefore we expect the results to be comparable. The lack of correspondence between true positive fusions, namely the 22 fusion events validated in the Body Map 2.0 in Frenkel-Morgenstern paper and results obtained with FusionMap on the same dataset in this paper, suggests that *ab-initio *chimera detection approaches are not sensitive enough to detect fusion genes in normal tissues. However, since chimeras detected by Frenkel-Morgenstern have a quite low representation in normal tissues, it is also possible that they were not sampled in the paired-end dataset for stochastic reasons.

## Conclusions

This paper highlights that specificity of state of the art tools for the identification of chimeras is affected at different degrees by read length and read quality scores of the RNA-seq dataset under analysis. Thus, it is important to carefully select the software on the basis of RNA-seq data features. In the specific case of detection of chimeras in normal tissues these fusion finder tools do not seem to provide results consistent with those obtained with a *knowledge-based approach *such as those reported by Frenkel-Morgenstern [7].

## Methods

### Fusion detection software

*MapSplice *[14] splits each read in a set of consecutive elements, then exon alignment is performed. MapSplice aligns any element not mapped in the previous step, using the knowledge resulting by other aligned elements. Splice junction quality is then assessed with two statistical measures: i) "anchor significance", given by an alignment that maximizes significance as a result of long anchors on the two sides of the splice junction, and ii) "entropy" calculated by the multiplicity of splice junction locations.

*FusionMap *[13] splits reads into smaller portions and it finds putative chimeras aligning these elements to genes annotated on genomic reference. The read alignment is based on GSPN algorithm [13], that provides a tolerance to mismatches of at most two bases. Seeds located at each side of an unmapped read are aligned to the reference. Chimeras are reported only if both seeds align, all chimeras having fusion boundaries distant less than 5 bp are combined and used to refine the position of junction boundary. Canonical splicing patterns are also used to refine the site of the fusion boundary, and false positives are removed using four filters. Reads are removed on the basis of their break point score; read-through fusions are discarded; chimera pseudo-reference are created and fusion without reads aligned to the pseudo-reference are removed; PCR artifact are also removed.

*FusionFinder *[11] divides reads into shorter elements and it detects chimeras aligning these fragments annotated genomic reference. The main differences with respect to FusionMap are related to alignment and filter implementation. Bowtie [22] is used to align fragments with respect to the coding reference transcriptome. Exons tagged as fusion elements go through some filtering steps to refine the results: (i) seeds mapping on the same gene are removed; (ii) pairs of reads mapping on the same chromosome but on opposite strands are discarded; (iii) pairs of reads mapped on genomic coordinates not associated to annotated genes are removed; and (iv) artifacts caused by sequence similarity are also discarded.

*deFuse *[10] uses reads pairs showing discordant alignments to detect putative chimeras essentially scoring putative fusions on the basis of fusion junction coverage and considering that shift between overlapping spanning reads must be consistent with the fragment length.

For each putative fusion, chimera boundaries are used to identify encompassing reads and to define fusion boundary at the nucleotide level. Paired-end reads aligning at a length that does not match with the expected distribution of sequenced fragments distance are discarded.

*FusionHunter *[12] aligns paired-end reads against a reference genome using Bowtie. The mapped reads are used to identify the fusion candidates, which are aggregated to generate a pseudo reference to detect junction-spanning reads. Unmapped reads are fragmented and aligned on the pseudo-reference. If one fragment is correctly aligned, the nearest canonical splicing junction is searched and the other part of the original read is aligned to this region. Chimeras made of two genes sharing significant homology are removed. Chimeras lacking at least two different paired-end reads supporting the fusion boundary are discarded. Furthermore reads mapping on the break point with less than 6 bp are removed as well as PCR artifacts and read-through events.

*TopHat-Fusion *[16] detects all reads mapping entirely within exons using Bowtie, and it creates a set of partial exons from these alignments. Pseudo-genes structures are then created, while unmapped reads are split into shorter elements and mapped on the genome. Chimeras are detected if reads fragments map in a consistent way with fusions (using TopHat [18] with relaxed parameters). Filtering is subsequently applied to eliminate (i) chimeras associated to multi-copy genes or repetitive sequences; (ii) reads mapping with less than 13 bp on either side of fusion; and read-through events.

TopHat-Fusion also keep track of contradicting reads, i.e. the reads mapping both on a single part of fusion and on fusion boundary.

### Data analysis

FusionHunter, FusionMap, FusionFinder, MapSplice, deFuse and TopHat-fusion were downloaded from the repository indicated in their papers and installed in adherence with the requirements indicated in their manual. All software tools were run with their default configuration. The analyses were performed on a 48 cores AMD server with 512 Gb RAM and 9 Tb HD, running linux SUSE Enterprise 11. Statistics and data parsing were executed using R scripting, taking advantage of the gplots-contributed R package http://cran.r-project.org/web/packages/gplots/ and Bioconductor [19] packages, i.e. Biostrings, org.Hs.eg.db, GenomicRanges and oneChannelGUI [20].

### Negative dataset

The negative dataset was generated using BEERS [17]http://www.cbil.upenn.edu/BEERS/, consisting of 70 million 100 paired-end reads (parameters: -readlength 100 -tlen 5 -tpercent 0.1). Since BEERS does not simulate Illumina quality scores, we attached to the 70 million reads the quality scores derived from 100 bp paired-end reads experiments run in our laboratory, to generate lib100_1 and lib100_2 fastq files. In addition from the 100 paired-end reads we generated a set of 2 × 75 nts (lib75_1 and lib75_2) and 2 × 50 nts paired-end reads (lib50_1 and lib50_2), removing 25 or 50 nts at the beginning of each read in the lib100_1 and lib100_2 fastq files, respectively. Negative datasets are available from the authors upon request.

### Positive dataset

FusionMap http://www.omicsoft.com/fusionmap/#Home developers provide a synthetic dataset of simulated paired-end RNA-Seq reads (~60,000 pairs of reads, 75 nt, fragment size = 158 bp). 50 fusions are represented, with a number of supporting pairs ranging from 9 to 8852. The sensitivity of each tool was calculated by dividing the number of chimeras detected by each tool with respect to the total number of chimeras in the positive dataset. The "false positive" behavior is instead reported directly as the number of chimeras detected that do not match any of the positive 50 chimeras.

### Fusion genes detected in the 75 bp Body map dataset

Frenkel-Morgenstern's paper [7] provided, as additional information, the list of chimeras detectable in the Body Map dataset (75 bp single-end reads) and the tissue in which they were detected. Furthermore, the paper also provided the fasta files for all the analyzed 7,424 putative human chimeric RNAs. Using R http://cran.r-project.org/ script we extracted the subset of 172 fusion events detected by Frenkel-Morgenstern in the Body Map 2.0. Each of the Frenkel-Morgenstern's 172 chimeras was manually blasted http://blast.ncbi.nlm.nih.gov/Blast.cgi against the human reference genome and we considered as a putative chimera only those characterized by a unique mapping on two different genomic locations. Moreover, we discarded all fusion events characterized by: i) having part of the sequence mapping on multiple genomic locations, ii) having the sequence mapping on the same genomic location, iii) having sequences mapping on more than two different chromosomal locations. Out of this filtering 22 fusion genes were left as putative chimeras (Table 3).

### Body Map 2.0

Illumina http://www.illumina.com has sequenced mRNAs derived from 16 normal tissues (Body Map 2.0: Adrenal gland, Adipose tissue, Brain, Breast, Colon, Heart, Kidney, Liver, Lung, Lymph Node, Ovary, Prostate, Skeletal Muscle, Testis, Thyroid, white Blood cells). These data are public available on the GEO database (GSE30611). Approximately 80 million reads for each tissue were provided as 75 bp single-ends reads (SE) or 50 nts paired-end reads (PE) datasets. SE and PE refer to the sequencing of one and both ends of a DNA fragment, respectively. The libraries used for sequencing were derived from poly-A selected mRNAs and generated by random priming. In case of PE, the average size of the sequenced fragment was approximately 300 bp. These datasets, due to the high number of reads provided, represent an ideal instrument for the identification of chimeras associated with normal tissue and to investigate chimeras tissue specificity [7].

## Competing interests

The authors declare that they have no competing interests.

## Authors' contributions

FL installed and setup fusions detection software and databases. MC and MB performed the comparison among fusion-finders. RAC collected data and generated negative dataset. FeC and SD revised the article and provided suggestions. RAC and FrC supervised the overall work.

## Supplementary Material

Additional file 1 **Chimeras detection in the negative datasets**. The number of reads distribution associated to false positive chimeras is shown for five fusion finders: THF1,2) TopHat-fusion with two different thresholds for the number of reads, FM1,2) FusionMap with two different thresholds for the number of reads, FF1,2) FusionFinder with two different thresholds for the number of reads, DF1,2) deFuse with two different thresholds for the number of reads, MS1,2) MapSplice with two different thresholds for the number of reads. FusionHunter is not shown since it does not detect false positive chimeras. Click here for file

## Declarations

The publication costs for this article were funded by FP7 EU Health Project "Next Generation Sequencing Platform for Targeted Personalized Therapy of Leukemia" (NGS - PTL) Grant Agreement n. 306242

This article has been published as part of *BMC Bioinformatics *Volume 14 Supplement 7, 2013: Italian Society of Bioinformatics (BITS): Annual Meeting 2012. The full contents of the supplement are available online at http://www.biomedcentral.com/bmcbioinformatics/supplements/14/S7
