# Supplementary figures and images for: State of art fusion-finder algorithms are suitable to detect transcription-induced chimeras in normal tissues?
Source: BMC Bioinformatics. 2013 Apr 22;14(Suppl 7):S2. doi: 10.1186/1471-2105-14-S7-S2 (PMC3633050; doi:10.1186/1471-2105-14-S7-S2)

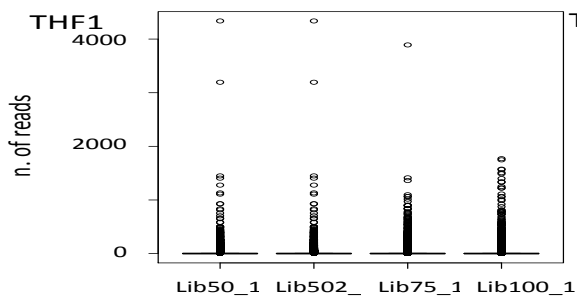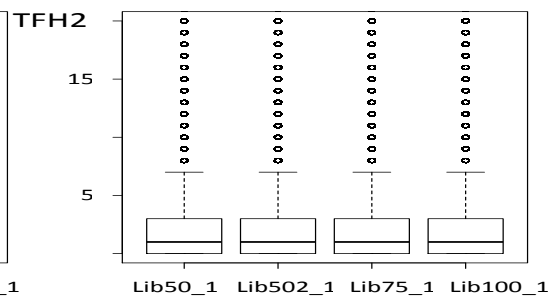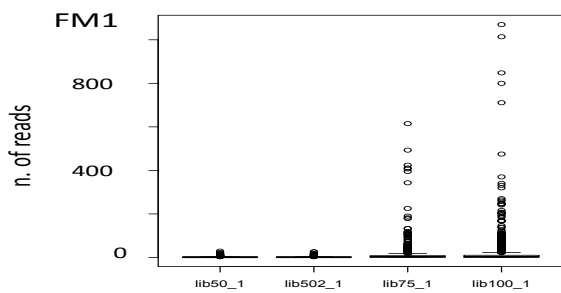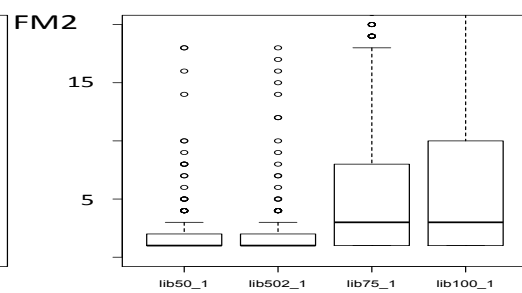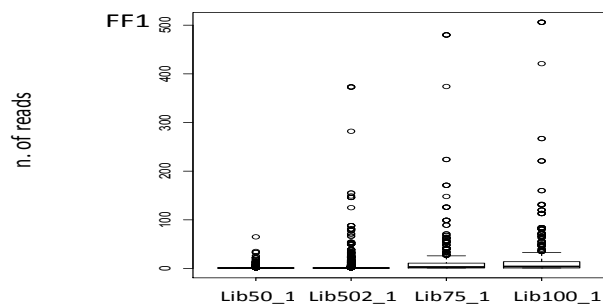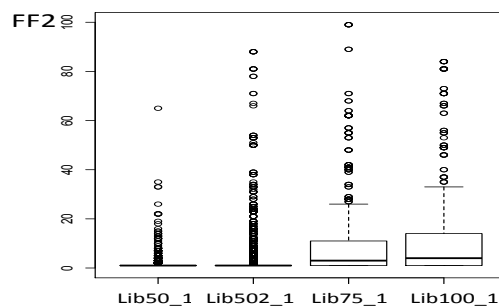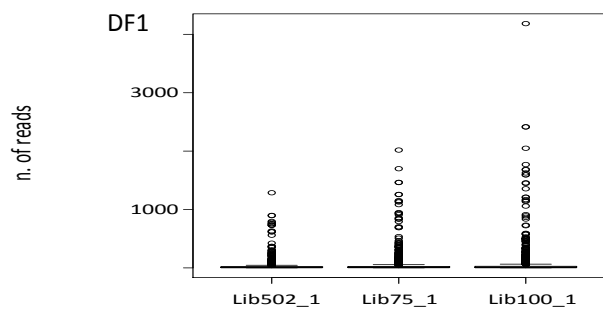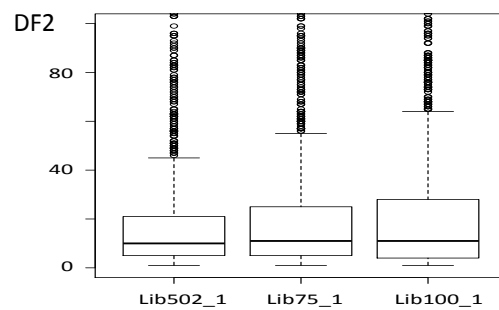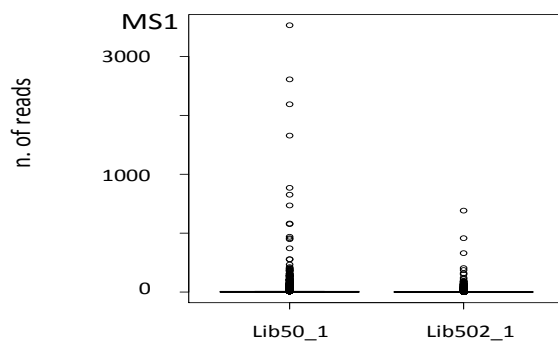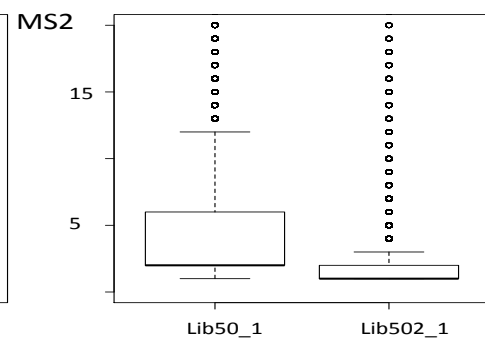

Supplement: Additional file 1 — Chimeras detection in the negative datasets. The number of reads distribution associated to false positive chimeras is shown for five fusion finders: THF1,2) TopHat-fusion with two different thresholds for the number of reads, FM1,2) FusionMap with two different thresholds for the number of reads, FF1,2) FusionFinder with two different thresholds for the number of reads, DF1,2) deFuse with two different thresholds for the number of reads, MS1,2) MapSplice with two different thresholds for the number of reads. FusionHunter is not shown since it does not detect false positive chimeras. [file 1471-2105-14-S7-S2-S1.pdf]
